# A Novel Affinity Engineered Anti-CD47 Antibody With Improved Therapeutic Index That Preserves Erythrocytes and Normal Immune Cells

**DOI:** 10.3389/fonc.2022.884196

**Published:** 2022-05-19

**Authors:** Youg R. Thaker, Ianne Rivera, Christophe Pedros, Alok R. Singh, Laura Rivero-Nava, Heyue Zhou, Barbara A. Swanson, Lisa Kerwin, Yanliang Zhang, J. Dixon Gray, Gunnar F. Kaufmann, Henry Ji, Robert D. Allen, Damien Bresson

**Affiliations:** ^1^Sorrento Therapeutics, Inc., San Diego, CA, United States; ^2^Janssen, San Diego, CA, United States; ^3^Turnstone Biologics, Center for Novel Therapeutics, La Jolla, CA, United States; ^4^Retired, Encinitas, CA, United States; ^5^Oncternal Therapeutics, San Diego, CA, United States

**Keywords:** CD47, immuno-therapy, T-cells, cancer, phagocytosis

## Abstract

Therapeutic blockade of the CD47/SIRPα axis by small molecules or monoclonal antibodies (mAbs) is a proven strategy to enhance macrophages-mediated anti-tumor activity. However, this strategy has been hampered by elevated on-target toxicities and rapid clearance due to the extensive CD47 expression on normal cells (“antigen sink”) such as red blood cells (RBCs). To address these hurdles, we report on the development of STI-6643, an affinity-engineered fully human anti-CD47 IgG_4_ antibody with negligible binding to normal cells. STI-6643 exhibited no hemagglutination activity on human RBCs at concentrations up to 300 µg/mL yet specifically blocked the CD47/SIPRα interaction. Of particular interest, STI-6643 preserved T cell functionality *in vitro* and showed significantly lower immune cell depletion *in vivo* in contrast to three previously published competitor reference anti-CD47 clones Hu5F9, AO-176 and 13H3. In cynomolgus monkeys, STI-6643 was well-tolerated at the highest dose tested (300 mg/kg/week) and provided favorable clinical safety margins. Finally, STI-6643 displayed comparable anti-tumor activity to the high-affinity reference clone Hu5F9 in a RAJI-Fluc xenograft tumor model as monotherapy or in combination with anti-CD20 (rituximab) or anti-CD38 (daratumumab) mAbs. These data suggest that STI-6643 possesses the characteristics of an effective therapeutic candidate given its potent anti-tumor activity and low toxicity profile.

## Introduction

Development of cancer confers several advantages to tumor cells over normal cells leading to their uncontrolled proliferation and evasion of apoptosis and phagocytosis. Several molecules involved in these escape mechanisms have been identified and targeted by cancer treatments (1). Less clear is the role of molecules associated with phagocytosis (2). Two immunoglobin family members CD31 and CD47 have been identified as the inhibitors of phagocytosis (2, 3). CD31 surface expression is detected on platelets, on most leukocytes, and is constitutively present on endothelial linings *in vivo* (4). CD47, on the other hand, is ubiquitously expressed and functions as a regulator of phagocytosis by interacting with its high-affinity ligand, signal regulatory protein alpha (SIRPα) (5) expressed on innate immune cells such as macrophages, dendritic cells, and neuronal cells (5–8). CD47 binding to SIRPα delivers an inhibitory “do not eat me” signal preventing phagocytic removal of healthy cells by the immune system (9–11). Therefore, CD47 represents an important inhibitory checkpoint preventing phagocytosis of healthy cells (9, 11, 12) and unwanted autoimmune attacks (13).

However, many cancer cells hijack this molecular pathway by expressing elevated levels of cell surface CD47 to avoid elimination by phagocytic immune cells. High CD47 expression is associated with poor clinical prognosis (9, 12, 14). Based on these observations, CD47 has become a prominent target in the field of cancer immunotherapy (5, 15–17). Consequently, a series of monoclonal antibodies (mAbs) blocking the CD47/SIRPα interaction are being evaluated clinically (18).

High CD47 expression on normal cells among which RBCs, platelets and lymphocytes express elevated levels of CD47 led to significant toxicities (mainly thrombocytopenia, anemia, neutropenia and lymphopenia) in the clinical trials with anti-CD47 mAb Hu5F9-G4 (18), which were mitigated by injecting patients with a low-priming mAb dose to occupy the RBC sink and induce compensatory erythropoiesis (19, 20). Recent studies have also described elevated CD47 levels in tissue-infiltrating NK cells and CD8^+^ T cells in esophageal carcinoma patients (21), but the impact of anti-CD47 treatments on lymphocyte and particularly effector T cells in patients has not been extensively documented. Although lymphopenia was observed in peripheral blood of approximately 35% Hu5F9-G4-treated patients (20) it remains unclear whether CD4^+^ and CD8^+^ tumor-infiltrating T cells are impacted by prolonged anti-CD47 treatments. This question remains crucial considering the importance of CD8^+^ T cells in boosting and maintaining anti-tumor responses upon anti-CD47 therapy as observed in preclinical animal models (19, 22–24). Thus, designing next generation CD47/SIRPα blocking antibodies with an improved safety profile and greater therapeutic window are needed.

Here, we describe the preclinical development of a fully human IgG_4_-S228P anti-CD47 antibody, named STI-6643 with clinically favorable properties. STI-6643 has been affinity-engineered to maintain the ‘on-target/on-tumor’ activity but reduce the ‘on-target/off-tumor’ binding to CD47. Consequently, STI-6643 displayed potent anti-tumor activity with negligible RBC and lymphocyte toxicities and was well-tolerated at doses up to 300 mg/kg in monkeys without the necessity of a priming dose. STI-6643 showed increased macrophage-mediated phagocytosis and anti-tumor activity when combined with anti-PD-L1, anti-CD20 or anti-CD38 mAbs. As such, one can envision use of STI-6643 in combination with tumor cell-specific opsonizing or lymphocytes-targeting immune-modulators for improved efficacy. Hence, STI-6643 possesses biophysical and functional properties to potentially become an important antibody in the field of CD47/SIRPα-axis blocking therapeutic agents and is set for clinical trials.

## Results

### Expression and Characterization of STI-6643 mAb

**Figure 1:**
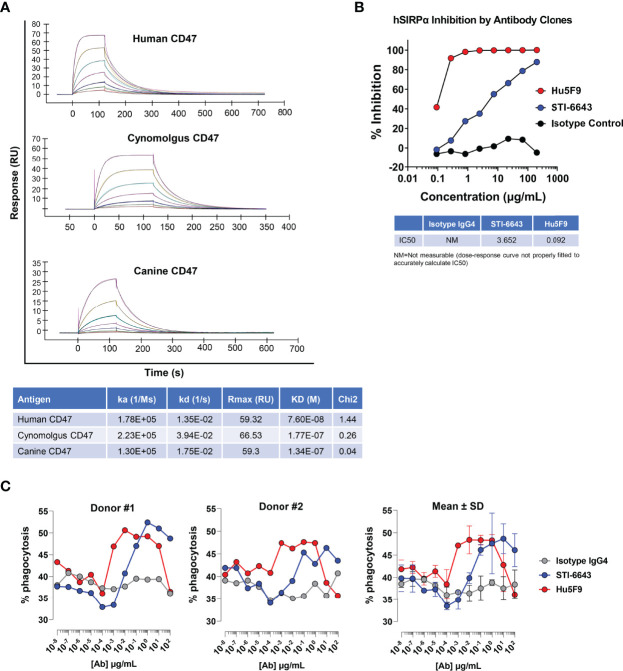
Kinetics and functional properties of human anti-CD47 clone STI-6643. **(A)** The kinetic binding parameters of STI-6643 to human (top panel), cynomolgus (middle panel) and canine (bottom panel) CD47 ligands were measured using the BIACORE and are given in the Table. **(B)** Blockade of the SIRPα/CD47 interaction by anti-CD47 clones STI-6643 (blue) and Hu5F9 (red) with a range of dose from 0.1 to 200 µg/mL. IC50 values are provided in the Table. NM, not measurable. **(C)** Phagocytosis of RAJI-GFP cells by CD14^+^ monocytes derived macrophages isolated from the PBMCs of two different donors (left and middle panel) in presence of STI-6643 (blue) and Hu5F9 (red) anti-CD47 antibodies or control isotype IgG_4_ (grey) antibodies. Cumulative data from both experiments are presented in the right panel. Data are representative of two **(A)**, two **(B)**, three **(C)** independent experiments.

Purified STI-6643 cGMP (current Good Manufacturing Practice) mAb was obtained as outlined in the Materials and Methods section. Anti-CD47 clone STI-6643 was affinity-engineered to exhibit reduced binding affinity towards its target with the hypothesis that avidity rather than affinity would drive target engagement and thus it would lower on-target off-tumor toxicity and improve bioavailability. **Figure 1A** shows that STI-6643 possesses a moderate binding affinity to human CD47 antigen with a K_D_ of 76 nM. The affinity was found to be even lower against the cynomolgus monkey and canine antigens (177 and 134 nM respectively).

We used reference anti-CD47 mAb clone Hu5F9-G4 (or Hu5F9) to compare the binding of STI-6643 on two human and two canine cancer cell lines (**Supplementary Figure 1A**). STI-6643 displayed similar dose-dependent binding to MDA-MB-231 and two canine osteosarcoma cell lines (OSCA-40 and OSCA-78) with an EC50 of ~ 30.0 µg/mL whereas binding to RAJI cells was slightly better (EC50 = 6.64 µg/mL). Binding of Hu5F9 was higher than STI-6643 on human cell lines (with saturating doses as low as 0.01 µg/mL) but was similar on canine cell lines (EC50 of ~ 24.6 µg/mL).

We next assessed the functionality of STI-6643, by analyzing its ability to block the SIRPα/CD47 interaction *in vitro* using CD47-presenting tumor cells and recombinant human SIRPα protein (**Figure 1B**). STI-6643 inhibited CD47/SIRPα binding in a dose-dependent manner with an IC50 of 3.65 µg/mL. The blocking activity was superior with high affinity reference mAb Hu5F9 (IC50 of 0.092 µg/mL) with saturating doses seen over 0.8 µg/mL of Hu5F9 vs no saturation seen in STI-6643 up to 200 µg/mL.

Blockade of the CD47/SIRPα pathway triggers the phagocytic activity of SIRPα-presenting macrophages towards CD47-expressing cells. Therefore, we performed a dose-response phagocytosis assay in presence of anti-CD47 mAb clones STI-6643 and Hu5F9. Both mAbs enhanced the phagocytosis of RAJI-GFP cells by CD14^+^-derived macrophages (**Figure 1C**, left and middle panels, average data presented in right panel). On average, Hu5F9 was more active than STI-6643 at low concentrations (from 0.0001 to 0.01 µg/mL) but both STI-6643 and Hu5F9 clones performed similarly at higher concentrations even if Hu5F9 activity dropped at concentrations above 10 µg/mL.

Since direct tumor cell killing activity has been reported for several anti-CD47 antibodies (25–28). STI-6643 killing activity was evaluated on CCRF-CEM tumor cells expressing high CD47 levels. Our results indicate that positive control anti-CD47 mAb CC2C6 (25) as well as reference clone Hu5F9 induced a concentration-dependent cell death of CCRF-CEM cells, but no activity was seen by STI-6643 (**Supplementary Figure 1B**).

### STI-6643 Shows Negligible RBC Binding, Hemagglutination and Toxicity

**Figure 2:**
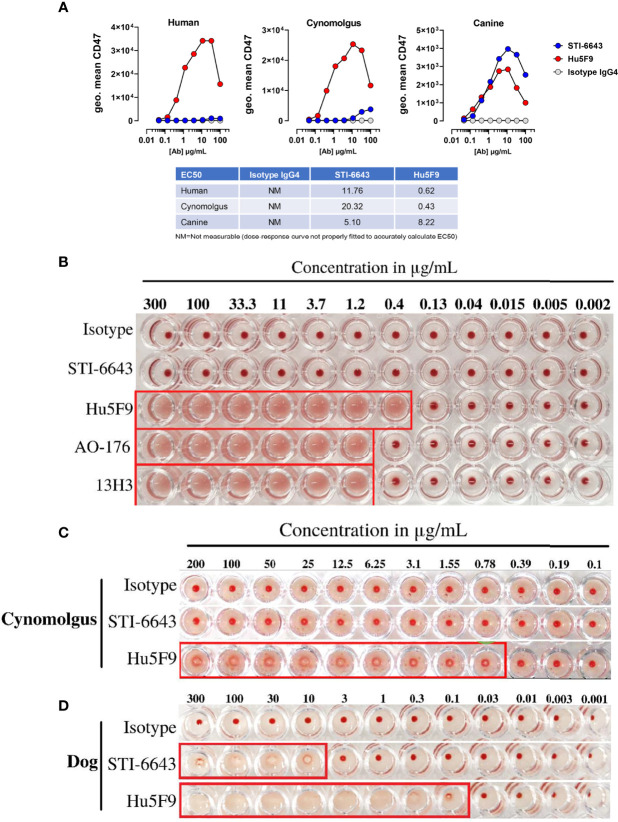
STI-6643 showed negligible RBC binding and hemagglutination compared to reference anti-CD47 clones **(A)** Binding of STI-6643 (blue), Hu5F9 (red) and isotype control (grey) antibodies to human (left), cynomolgus monkey (middle) and canine (right) RBCs. **(B)** Hemagglutination activity of anti-CD47 mAbs clones STI-6643, Hu5F9, 13H3 and AO-176 or isotype control on human RBCs at 20 hours post incubation at 37°C. Hemagglutination of cynomolgus monkey **(C)** and dog **(D)** RBCs induced by STI-6643 and Hu5F9. A matching isotype IgG_4_ antibody was used as negative control. Data are representative of one **(A)**, two **(B)**, three **(C, D)**, independent experiments.

Since one of the most obvious toxicities associated with CD47-targeting antibodies is the partial depletion of RBCs leading to anemia, we compared the binding ability of anti-CD47 clones STI-6643 and Hu5F9 to RBCs isolated from different species. STI-6643 showed negligible binding to human and cynomolgus RBCs as compared to the reference clone Hu5F9 (**Figure 2A**). STI-6643 bound slightly better to canine RBCs (EC50 = 5.1 µg/mL) than human and cynomolgus RBCs (EC50 = 11.76 and 20.32 µg/mL respectively) whereas clone Hu5F9 showed reduced binding capacity to canine (EC50 = 8.22 µg/mL) versus human or cynomolgus RBCs (EC50 = 0.62 and 0.43 µg/mL respectively).

Next, we measured hemagglutination of human, cynomolgus monkey and dog RBCs in presence of STI-6643 and three reference clones (Hu5F9, AO-176 and 13H3). As shown **Figures 2B, C**, STI-6643 did not induce hemagglutination of human or cynomolgus RBCs at concentrations up to 300 µg/mL. In stark contrast, all reference clones demonstrated hemagglutination activity in the same conditions at concentrations higher than 1.2 µg/mL while Hu5F9 hemagglutinated as low as 0.4 µg/mL (**Figures 2B, C**). RBCs isolated from canine blood was the only species against which STI-6643 showed signs of hemagglutination (**Figure 2D**) correlating with the increased binding to canine RBCs (**Figure 2A**). However, despite similar binding of Hu5F9 and STI-6643 to dog RBCs, Hu5F9 hemagglutination activity was 100-fold superior (**Figures 2A, D**).

**Figure 3:**
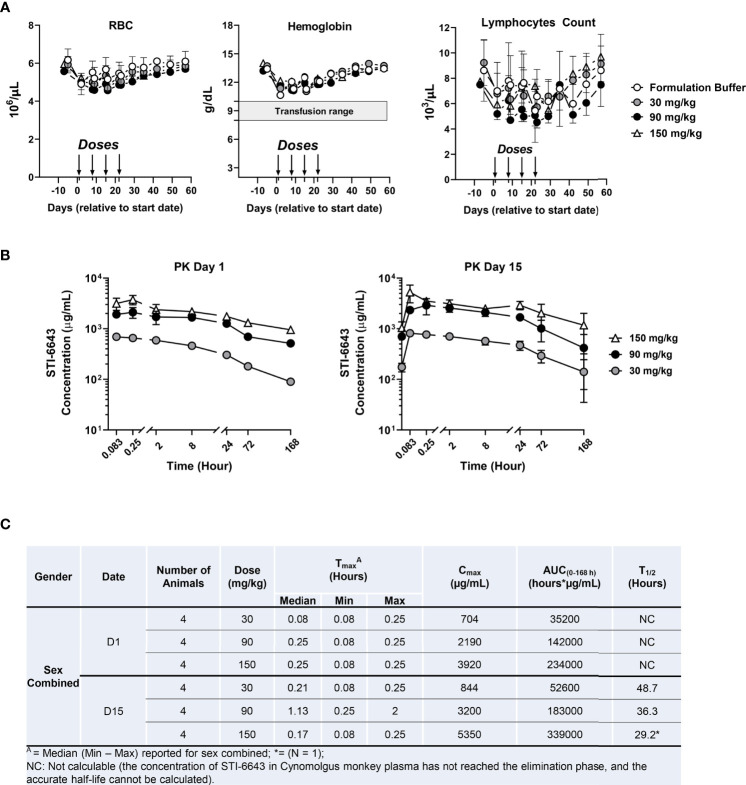
STI-6643 demonstrates high safety profile in non-human primate toxicokinetic study. Individual cynomolgus monkeys were administered intravenously on days 0, 7, 14 and 21 with STI-6643 at the indicated doses or the same volume of formulation buffer (marked by arrows). The hematological parameters of individual monkeys were monitored for 9 weeks. **(A)** The hemoglobin levels, the RBC and lymphocytes counts were measured overtime. The shaded bar indicates the range of hemoglobin that might trigger transfusion in humans. **(B)** Serum STI-6643 levels were determined after a single infusion (PK day 1) or after the third infusion (PK day 15) at the indicated doses. **(C)** Pharmacokinetic parameters in cynomolgus monkeys dosed in panel **(B)** T_max_: time of C_max_, C_max_: maximum observed concentration, AUC_(0-168h)_: area under the curve and T_1/2_: half-life. Data are given as a mean ± SD.

To assess tolerability and ‘on-target/off-tumor’ toxicity of STI-6643, a dose-finding study was conducted in non-human primates (NHP). STI-6643 treatment was well-tolerated since no statistically significant difference was measured between the ‘formulation buffer’ control- or STI-6643-treated groups at doses up to 150 mg/kg/dose for hemoglobin concentration, RBC and lymphocyte counts (**Figure 3A**). The no-observed-adverse-effect level (NOAEL) was 150 mg/kg/dose for up to four doses.

Pharmacokinetic (PK) analysis indicated T_max_ was generally observed at 0.08 and 0.25 hours post treatment on day 1 (**Figures 3B, C**). When estimable, individual T_1/2_ ranged from 29.2 to 48.7 hours after the third injection on day 15., Based on mean C_max_ and AUC_(0-168)_ values, systemic exposure to STI-6643 increased in an approximately dose proportional manner in both sexes. Accumulation of STI-6643 was not observed on day 15 when compared to the exposure on day 1.

In a follow-up study, we investigated the safety of high dose (300 mg/kg/dose) treatment (**Supplementary Figure 2**). Even at 300 mg/kg/dose, STI-6643 was well-tolerated, RBCs counts and hemoglobin concentration were rarely found significantly reduced when compared to formulation buffer treated animals (**Supplementary Figure 2A**). On average, the hemoglobin concentration never dropped below 10 g/dL (a hemoglobin concentration which might trigger transfusion in humans). The lymphocytes counts were never significantly different between STI-6643-treated and control groups in both males and females. No test article-related abnormalities in the clinical or physical were noted in males or females in the STI-6643 treated group (data not shown). Furthermore, there was no statistically significant difference of C_max_ or AUC_(0-168 h)_ between two sexes; C_max_ and AUC_(0-168 h)_ of STI-6643 increased with the dose levels but no significant accumulation of STI-6643 was observed after repeated dosing (**Supplementary Figures 2B, C**). Based on these results, the highest non-severely toxic dose (HNSTD) was determined to be 300 mg/kg.

### STI-6643 Shows Potent Preclinical Anti-Tumor Activity in Animal Models

**Figure 4:**
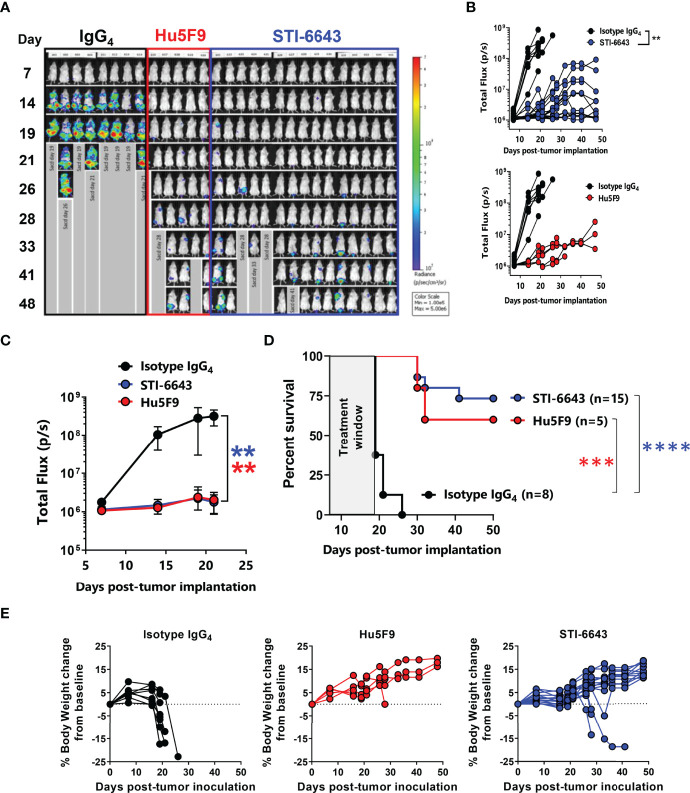
STI-6643 treatment shows comparable anti-tumor activity to reference clone Hu5F9 in the RAJI-Fluc disseminated xenograft model **(A-C)** Systemic tumor growth was measured by bioluminescence until day 48 post tumor cell implantation. Flux images recorded weekly **(A)** and bioluminescence flux quantification **(B)** of animals treated at 30 mpk/injection for 6 doses over a 2-week period with isotype (8 mice, black), STI-6643 (15 mice, blue) or Hu5F9 (5 mice, red). Average flux data **(C)** and survival plots **(D)** from each treatment group. **(E)** Percent body weight change from baseline for isotype (left), Hu5F9 (middle) and STI-6643 (right) monitored weekly until study termination. Data are representative of 4 independent experiments and shown as means ± SEM **(C, D)**; Statistical significance assessed by two-way ANOVA **(B, C)** and log-rank Mantel–Cox test **(D)**. **p < 0.01, ***p < 0.005, ****p < 0.0001.

To determine the anti-tumor activity of STI-6643, we used the disseminated RAJI-Burkitt’s lymphoma tumor xenograft model in immunodeficient Fox Chase/SCID mice. Tumor-bearing mice were treated with either a human IgG_4_ mAb isotype control or anti-CD47 clones STI-6643 or Hu5F9 at 30 mg/kg (mpk) for 6 doses over a 2-week period. Systemic tumor growth was measured by bioluminescence revealing a significant anti-tumor activity induced by anti-CD47 mAbs when compared to isotype control (**Figures 4A–C**) correlating with a prolonged survival of STI-6643 (73%) and Hu5F9 (60%) treated mice as compared to isotype (0%) treated mice (**Figure 4D**). Both anti-CD47 mAbs clones showed similar anti-tumor activity and no statistical difference in survival rate was observed between STI-6643 and Hu5F9-treated animals. Body weights of isotype treated mice significantly dropped around day 20 due to high tumor burden (**Figure 4E**, left panel). In contrast, most animals treated with either STI-6643 or Hu5F9 maintained healthy body weights (**Figure 4E**, middle and right panels).

**Figure 5:**
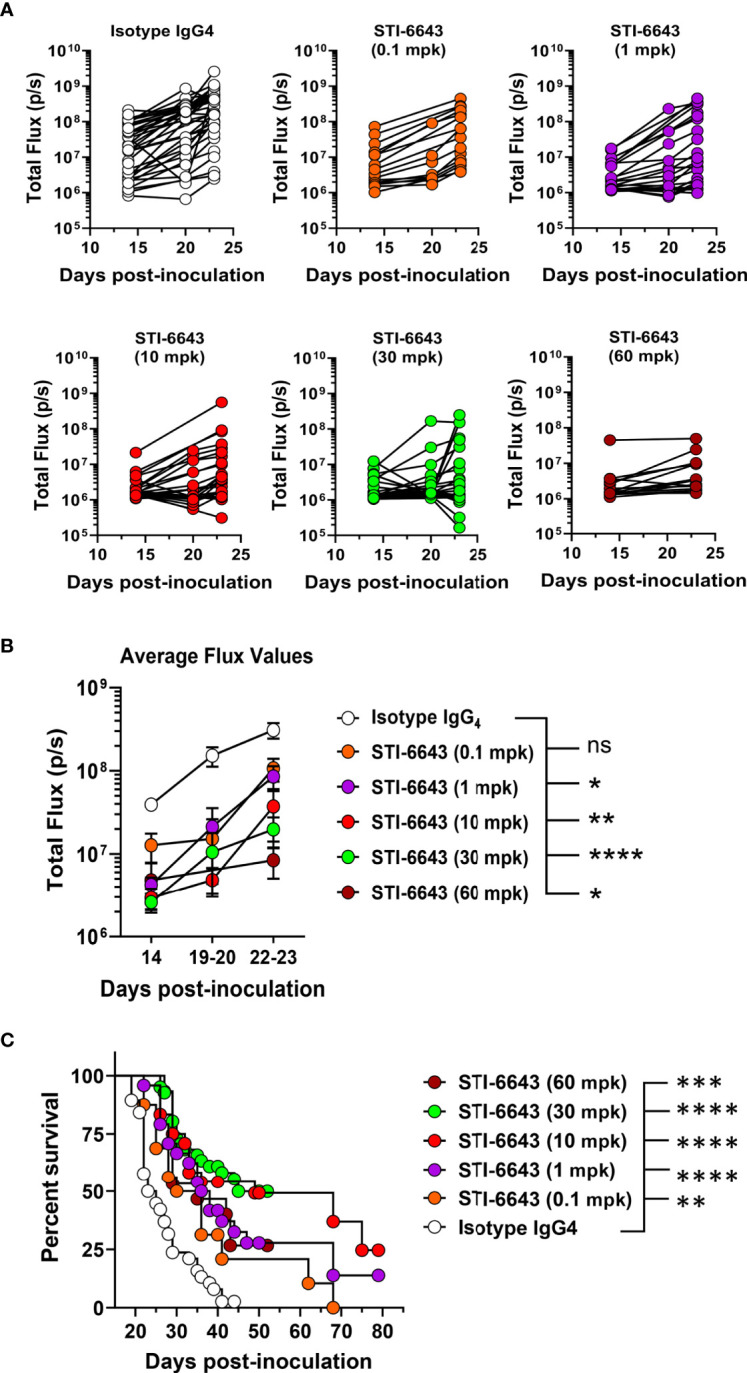
Dose titration of anti-tumor activity of STI-6643 in the hematologic RAJI xenograft model. **(A, B)** Systemic tumor burden measured by bioluminescence in each group treated with STI-6643 or isotype IgG_4_ (at various doses ranging from 0.1 to 60 mg/kg dose) showing total flux values from either **(A)** individual mice or **(B)** average data (means ± SEM). **(C)** Grouped analysis of survival data from 5 independent experiments comparing STI-6643-treated groups at different doses with the isotype control group. Data are pooled from four independent experiments **(A, B)** or five **(C)** independent experiments. Number of animals used in each treatment group in each experiment ranged from 7-15 animals/group. A statistical analysis was conducted by comparing data from the anti-CD47 treatment groups to the isotype IgG_4_ treated group **(B, C)**. Statistical significance assessed by two-way ANOVA **(B)** and log-rank Mantel-cox test **(C)**; (not significant [ns], p > 0.05; *p ≤ 0.05; **p ≤ 0.01; ***p ≤ 0.001; ****p ≤ 0.0001).

We also evaluated the minimal efficacious dose of STI-6643 in mice receiving doses ranging from 0.1 to 60 mpk (**Figure 5** and **Supplementary Figure 3**) thrice a week for two consecutive weeks. Results indicated that at least 10 mpk thrice a week for 2 consecutive weeks was usually required to inhibit systemic tumor expansion and prolong survival when more aggressive tumor growth kinetics were observed (**Supplementary Figure 3**). However, when tumor development was less aggressive, doses as low as 1 mpk were sufficient to produce significant anti-tumor activity (**Figure 5B**) and survival (**Figure 5C**).

### Combination of STI-6643 and Tumor-Targeting IgG_1_ mAbs With Fc Receptor-Dependent Effector Function

Several published preclinical studies have demonstrated that anti-CD47 antibody treatment synergizes with tumor targeting IgG_1_ antibodies (among which anti-CD20, anti-PD-L1, anti-CD38 or anti-EGFR mAbs) by combining Fcγ receptor (FcγR)-dependent and FcγR-independent stimulation of phagocytosis as well as other FcR-dependent mechanisms such as antibody-dependent cell cytotoxicity (ADCC) (15, 18, 20, 26–28) leading to several ongoing clinical trials.

First, we evaluated *in vitro* the phagocytic activity of STI-6643 in combination with anti-CD20 (rituximab) or anti-PD-L1 (avelumab) mAbs (**Supplementary Figure 4A**). The combination of STI-6643 and rituximab induced superior phagocytic activities of CD14^+^ monocytes derived macrophages on RAJI-GFP cells at suboptimal doses of 2.5 and 5 ng/mL with an increase of 106% and 64% respectively as compared to single treatments. Similarly, STI-6643 plus avelumab combination demonstrated higher phagocytosis of the MDA-MB-231 breast tumor cell line when both drugs were combined as compared to single agent treatments (**Supplementary Figure 4B**).

**Figure 6:**
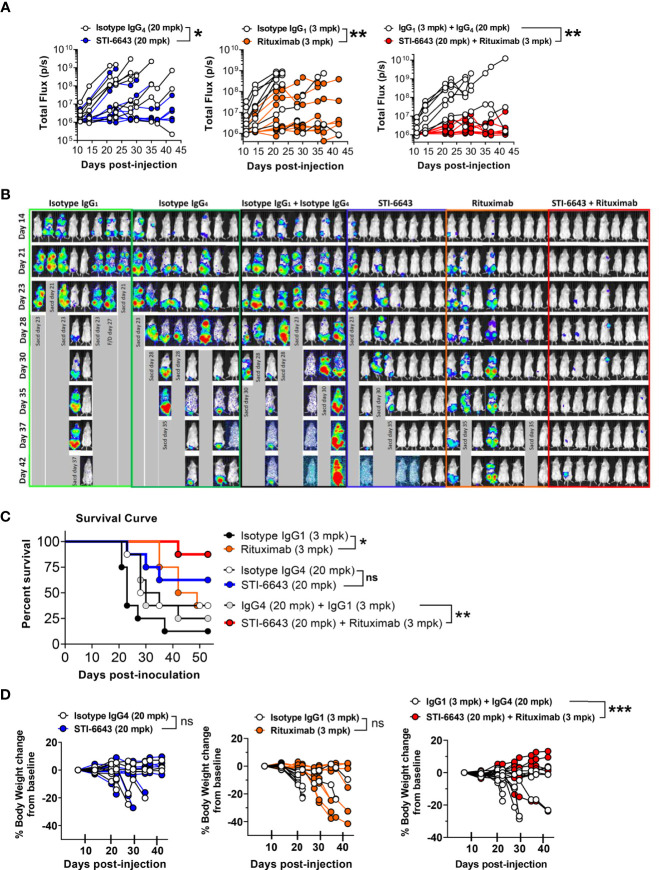
Addition of STI-6643 to Rituximab therapy foster anti-tumor activity in a Burkitt’s lymphoma xenograft model. **(A)** Systemic tumor burden measured by whole body bioluminescence (total flux) of individual mice in mice treated with STI-6643 and rituximab alone or in combination. Statistical analysis was conducted by comparing data from the anti-CD47 and Rituximab mono or combo-treatment groups to the isotype IgG_4_ treated groups. **(B)** Images of whole-body luminescence recorded from individual mice. **(C)** Survival data displayed for each treatment group. **(D)** Percent body weight change from baseline for each group until study termination of the study. Data are representative of two independent experiments. Group size is 8 animals per treatment condition. Statistical significance assessed by two-way ANOVA **(B)** and log-rank Mantel-cox test **(C)**; (not significant [ns], p > 0.05; *p ≤ 0.05; **p ≤ 0.01; ***p ≤ 0.001.

In subsequent studies, we investigated these combination treatments in xenograft RAJI-Fluc preclinical model. Our data demonstrate an improved anti-tumor activity when rituximab and STI-6643 were combined as compared to monotherapy (**Figures 6A, B**) which correlated with a significantly prolonged survival (**Figure 6C**). Endpoint survival was significantly higher (88% for the STI-6643 and rituximab combination) as compared to monotherapies (63% for STI-6643 and 38% for rituximab) and 13% for the IgG_1_ isotype-treated group (**Figure 6C**). No adverse events or toxicity due to STI-6643 or rituximab mAbs treatment were noted throughout the course of the study (**Figure 6D**). In a subsequent study, we also observed an increased survival rate when STI-6643 was combined with daratumumab as compared to the daratumumab monotherapy (**Supplementary Figure 5**). Overall, our data showed the potential of STI-6643 to become a strong candidate for multiple combination therapies.

### STI-6643 Preserved Immune Cells Survival and Functionality *In Vitro*

**Figure 7:**
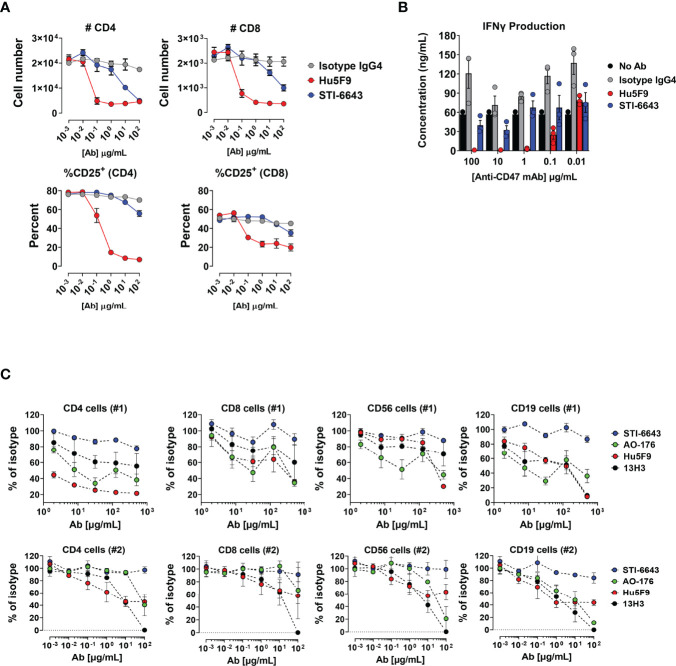
Anti-CD47 clone STI-6643 preserved T cells survival and functionality *in vitro*. **(A)** Data from SEB assay performed in presence of anti-CD47 clones STI-6643 (blue) and Hu5F9 (red) or isotype control. The number of CD4^+^ and CD8^+^ T cells as total cell counts (top panels) and frequencies of CD25^+^ activated T cells (bottom panels) recovered at the end of the assay is presented. **(B)** Quantification of IFN-γ from culture supernatants of SEB assay at day 3. Data are representative of one experiment. **(C)** Frequency (percent of isotype control) of live immune cells recovered from 3-way MLR assays in presence of anti-CD47 (clones STI-6643, Hu5F9, AO-176 and 13H3) or isotype IgG_4_ mAbs incubated at concentrations ranging from 2 to 500 µg/mL (#1; top panel) or 0.001 to 100 µg/mL (#2; bottom panel) without additional stimulation. Data were pooled from at least two independent experiments for each condition and cell numbers normalized to isotype values. Mean ± SEM shown.

NHP toxicity studies above demonstrated that STI-6643 treatments did not affect lymphocytes count. This observation is particularly important considering potential combination therapies targeting ‘healthy’ immune cells with immunomodulators to boost adaptive anti-tumor immunity. Hence, we conducted a series of experiments to investigate the impact of anti-CD47 clones on human T, B and NK lymphocytes *in vitro* and *in vivo*. First, we compared the activity of STI-6643 and reference clone Hu5F9 on T cell depletion and activation in a super antigen stimulation assay using Staphylococcal Enterotoxin B (SEB). Hu5F9 induces a dose-dependent reduction of total CD4^+^ and CD8^+^ T cells (**Figure 7A**, top panels) and lowered frequency of activated CD25^+^ T cells (bottom panels). STI-6643, on the other hand, had a minimal impact on T cell frequencies when compared to Hu5F9, with higher effects observed in the CD4^+^ T cell compartment. Of note, STI-6643 concentrations 1,000-fold superior to Hu5F9 were required to observe the similar activities between two clones (100 versus 0.1 µg/mL, respectively). In addition, STI-6643 had a minor impact on IFNγ secretion as compared to Hu5F9, suggesting that STI-6643 was able to preserve T cell functionality (**Figure 7B**).

Subsequently we conducted a series of 3-way mixed lymphocyte reaction (MLR) assays to address the same question in a more physiologic stimulation context. PBMCs from 3 different donors were mixed in equivalent proportions and incubated in the presence of anti-CD47 mAbs clones STI-6643, Hu5F9, AO-176, 13H3 or isotype IgG_4_ without additional stimulation. The number of CD4^+^, CD8^+^, CD19^+^ and CD56^+^ cells recovered at the end of the 6-day incubation period are shown when mAbs were tested at a concentration range of 2 to 500 µg/mL (**Figure 7C**; upper panels) or 0.001 to 100 ug/mL (**Figure 7C**; lower panels). The presence of anti-CD47 mAb Hu5F9 reduced, in a dose-dependent manner, the number of CD4^+^, CD19^+^ and CD56^+^ cells recovered. Both AO-176 and 13H3 reference mAbs also reduced the numbers of immune cells recovered but were not as potent as Hu5F9. In contrast to the reference clones, STI-6643 preserved the number of healthy T, B and NK immune cells.

### STI-6643 Preserved Immune Cell Survival and Functionality *In Vivo*

**Figure 8:**
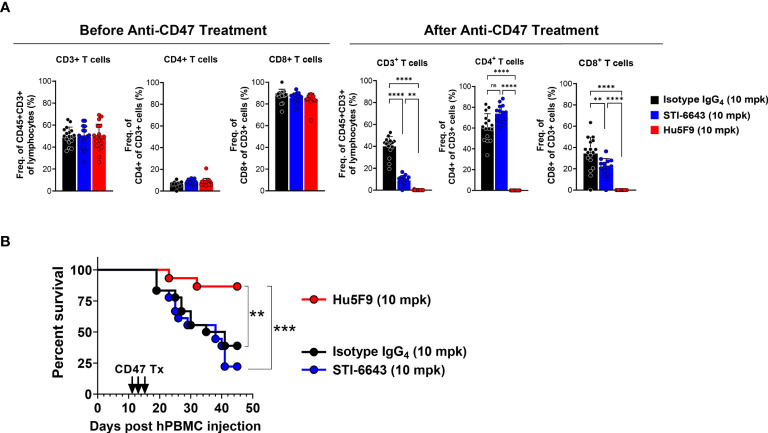
STI-6643 significantly preserved T cell function in a xenograft GVHD model. Immuno-deficient mice were engrafted with human PBMCs and subsequently treated with three doses (on days 11, 13 and 15) of either anti-CD47 clones STI-6643 or Hu5F9 or isotype IgG_4_ control antibodies at 10 mg/kg/dose. **(A)** Analysis of circulating T cells showing frequency of CD3^+^, CD4^+^ and CD8^+^ T cells before (d10, left panels) and after (d19, bottom panels) treatment. **(B)** Kaplan-Meier survival graph of mice with different treatments (n = 9 mice/group). Statistical significance assessed by one way ANOVA **(A)** and log-rank Mantel-cox test **(B)**; (not significant [ns], *p ≤ 0.05, **p ≤ 0.01, ***p ≤ 0.001, ****p ≤ 0.0001).

Next, we evaluated the impact of STI-6643 and Hu5F9 on human T cell function in a xenograft graft-versus-host disease (GVHD) model. Immunodeficient mice engrafted with human PBMCs were treated with three doses (on days 11, 13 and 15 post PBMC injection) of STI-6643 or Hu5F9 or isotype IgG_4_ control antibodies at 10 mpk. Prior to treatment (day 10), the peripheral blood of the mice contained ~50% of CD3^+^ T cells of CD45+ lymphocytes (**Figure 8A**, left panels). A skewed CD4:CD8 ratio was likely due to the PBMC donor. Interestingly, Hu5F9 treatment led to a strong (and almost complete) depletion of human T cells, directly impacting the development of GVHD (**Figure 8B**). In contrast, STI-6643 treatment led to a modest (~50%) decrease in CD3^+^ T cells as compared to isotype IgG_4_ (**Figure 8A**, right panels). However, STI-6643 treatment re-established a more physiological CD4:CD8 ratio of 2:1 and remained functional as indicated by their ability to induce GVHD with the same kinetics as the isotype control-treated mice (**Figure 8B**).

In a follow-up experiment, we evaluated the activity of STI-6643 and reference anti-CD47 clones AO-176 and Hu5F9 in solid tumor-bearing humanized mice (**Supplementary Figure 6**). Even if none of the antibodies showed any significant anti-tumor activity in this model, treatment with competitor clones AO-176 and Hu5F9 significantly reduced the numbers of all profiled circulating and tumor infiltrating immune cells after one week of treatment as compared to STI-6643. Hu5F9-was more a potent depleter than AO-176, especially of activated T cell populations (CD25^+^CD4^+^ and CD25^+^CD8^+^). Altogether, our data suggest that unlike reference anti-CD47 mAbs, STI-6643 showed minimal impact on T, B or NK cell depletion, preserving a pool of functional adaptive immune cells.

## Discussion

A series of immuno-modulators targeting the CD47/SIRPα pathway are currently being developed preclinically or evaluated in clinical trials. These checkpoint inhibitors blocking either side CD47/SIRPα pathway remove the brakes on macrophages to reinvigorate their phagocytic activity. This ‘regain-of-function’ improves the clearance of cancer cells but also leads to unwanted removal of the anti-CD47-sensitive normal cells such as erythrocytes or platelets, causing transient anemia or thrombocytopenia in treated patients (20, 26). In addition, early studies revealed a role for anti-CD47 mAbs in the priming of an adaptive immune response leading to enhanced anti-cancer activity by expanding tumor-specific cytotoxic T cells (23, 29, 30). The ubiquitous expression of CD47 on healthy tissues is also suspected to act as an antigen sink which can directly impact the bioavailability of CD47-targeted drugs.

Here, we report on the development of an affinity-engineered anti-CD47 IgG_4_ mAb named STI-6643 that was selected by using the G-MAB™ technology (31). STI-6643 has lower binding affinity to human CD47 than reference clones Hu5F9, AO-176 or 13H3. Our data indicate that this strategy improved the tumor selectivity by reducing the interaction with CD47 expressed on normal cells. As a result, STI-6643 did not hemagglutinate human and cynomolgus RBCs. In contrast, under identical assay conditions all three references clones agglutinated human RBCs at doses higher than 1.2 µg/mL. It was previously reported that AO-176 was not agglutinating RBCs at doses up to 1 mg/mL *in vitro* (8); however, the assay conditions slightly differed since PBS1X containing 1 mM ethylenediaminetetraacetic acid (EDTA) was used as buffer and the antibody was incubated for only 4 hours at 37°C. Unexpectedly, STI-6643 was found to hemagglutinate canine RBCs at intermediate to high concentrations likely due to its higher binding on canine cells (**Figure 2A**). Since CD47 amino acid sequence between canine and human share 66% identity (79% similarity) (32), one may assume that the epitope recognized by STI-6643 on human CD47 might be distinct than canine CD47. If this hypothesis is correct, then such changes improved STI-6643 binding to canine CD47 while it negatively impacted the binding of Hu5F9 as previously reported (32). Curiously, even if both mAbs displayed similar binding profiles to canine CD47 the stronger agglutination activity of Hu5F9 on human RBCs was still conserved on canine RBCs (2-log difference between STI-6643 and Hu5F9), suggesting additional mechanisms at play.

Along with its RBCs sparing property, STI-6643 also preserved human lymphocytes viability and T cell functionality *in vitro* in stark contrast to Hu5F9. A similar trend was observed *in vivo* in xenograft GVHD model. In contrast to Hu5F9, which drastically reduced circulating T cells numbers and consequently increased mouse survival by limiting GVHD, repeated STI-6643 treatments although reducing the total circulating CD3^+^ T cells had no impact on the GVHD development. We hypothesize that STI-6643 treatment affected naïve T cells to a greater extent than CD25^+^ activated/effector T cells as observed *in vivo* (**Supplementary Figure 6**) where the number of total CD4^+^ or CD8^+^ T cells was slightly reduced (when compared to isotype control) while CD4^+^CD25^+^ or CD8^+^CD25^+^ remained unaffected. Therefore, in GVHD model removal of naïve T cells (STI-6643 scenario) would have a minimal impact on GVHD kinetics whereas depletion of total T cells including CD25^+^ effector T cells (Hu5F9 scenario) would significantly slow down GVHD progression.

Residual STI-6643 activity seen on human T cells in mouse xenograft models was not recapitulated in toxicity studies conducted in NHP. Our data demonstrated STI-6643 was well-tolerated with minimal impact on RBCs and lymphocytes counts (including T cells) at doses up to 300 mg/kg/dose. STI-6643 had no significant on-target/off-tumor toxicities and a good PK profile without needing low-dose priming sometimes required for competitor antibodies to mitigate the anemia and reduce CD47 expression on circulating erythrocytes (19).

While sparing normal cells, STI-6643 also demonstrated specific dose-response binding activity to cancer cells usually expressing higher CD47 densities at the cell surface thus promoting increased avidity. Although STI-6643 functional activities (CD47/SIRPα blocking, phagocytosis) were found negligible at concentrations lower than 0.1 µg/mL, the antibody was as potent as Hu5F9 at concentrations above 200 µg/mL which are usually observed in circulating blood upon anti-CD47 infusions at clinically relevant doses (8, 19, 20). *In vivo*, STI-6643 and Hu5F9 were found equally potent when their circulating concentration was between 100 and 200 µg/mL (data not shown). In clinical settings, even if complete responses (CRs) were reported in some anti-CD47 trials, CRs were mostly below 50% and only affecting a sub-population of tumor patients. These observations were the rationale for using anti-CD47 mAbs in combination with other tumor targeting agents to improve on efficacy. For instance, it has been reported that in Non-Germinal Center B-cell Diffuse Large B-cell Lymphoma patients, high CD47 expression correlated with reduced overall survival upon rituximab and chemotherapy treatment suggesting that CD47 co-treatment with rituximab could be beneficial in those patients (14). Indeed, this strategy was found effective both in animal models and early clinical trials (14, 15, 27, 32). Supporting these observation, we also noted that STI-6643 had enhanced *in vivo* anti-tumor effects when co-administered with rituximab or daratumumab in the disseminated RAJI-Fluc xenograft tumor model.

Altogether, our data suggest that by engineering the binding affinity of STI-6643 we generated a mAb with a unique set of bio-properties including potent anti-tumor activities associated with negligible impact on normal lymphocytes and RBCs. These characteristics of STI-6643 should not only improve the safety profile but could concomitantly provide significant benefits for cancer patient treated with STI-6643 alone or in combination with other immune modulators.

## Materials and Methods

### Cloning and Expression of Fully Human Anti-CD47 IgG4-S228P Antibody Clone STI-6643

Briefly, codon optimized heavy and light chain genes of anti-CD47 IgG4-S228P clone STI-6643 were cloned into Lonza vectors pXC-18.4 and pXC-17.4, respectively, and the two vectors combined into a double gene vector before transfection. Clone selection procedure is described in Supplementary Material and Methods section.

STI-6643 mAb was produced under cGMP conditions using the master cell bank, purified with three chromatography steps: Protein A affinity chromatography, cation exchange chromatography and mixed-mode interaction chromatography to obtain high quality product for IND-enabling studies and future clinical trials. Nanofiltration was used to remove virus particles. The antibody was formulated with FDA-approved excipients and buffers to make the final product.

### Animals

Six to eight week-old female Fox Chase/SCID mice (CB17/Icr-*Prkdc^scid^*/IcrIcoCrl; strain# 236) and NSG-Tg (hIL-15) (JAX, NOD.Cg-Prkdcscid Il2rgtm1Wjl Tg(IL15)1Sz/SzJ, stock No. 030890) were obtained from Charles River or Jackson Labs and housed at the Sorrento Therapeutics animal facility in San Diego, California, USA. Experiments are conducted under ACUP# EB16-057 and EB17-010-018 in accordance with Explora BioLabs IACUC guidelines.

### Cell Lines

RAJI and CCRF-CEM cell lines were obtained from ATCC and maintained in complete RPMI-1640 media with GlutaMax supplemented with 10% FCS and 1% Penicillin/Streptomycin (P/S). MDA-MB-231 (ATCC) was maintained in DMEM supplemented with 10% FCS and 1% P/S. Both RAJI and MDA-MB-231 cells were transduced with a firefly luciferase (Fluc)-expressing lentivirus (Gentarget; Cat. LVP437) and selected with 0.5 µg/mL Puromycin to generate Fluc versions. Canine Osteosarcoma cell lines (OSCA-40 and OSCA-78) cells were obtained from Kerafast (Cat. EMN003 and EMN004, respectively) and expanded in DMEM, 10mM HEPES, 10%FBS and 100 µg/mL Primocin.

### Flow-Based SIRPα/CD47 Blockade Assay

20,000 CCRF-CEM cells per well were plated in U-bottom 96-well plate and incubated for 15 minutes at 37°C with anti-CD47 clones Hu5F9 or STI-6643 or Isotype IgG_4_ control mAbs at concentrations ranging from 200 to 0.09 µg/mL. Without washing, 50 µL of purified SIRPα-Fc fusion protein (R&D Systems; Cat. 4546-SA-050) diluted at 0.4 µg/mL in FACS buffer at 37°C was added to each well and the incubation was prolonged for another 20 minutes at 37°C. Cells were then washed three times and resuspended in FACS buffer. To reveal the binding of SIRPα-Fc fusion protein to CCRF-CEM cells, a PE-conjugated anti-SIRPα antibody (R&D Systems, Cat. FAB4546P) was used at 5 µL/well in 70 µL of FACS buffer for 20 minutes at RT in the dark. Cells were washed twice and resuspension in FACS buffer and analyzed immediately by flow cytometry.

### Phagocytosis Assays

CD14^+^ cells were isolated from human PBMCs using anti-human biotin CD14^+^ antibody (BioLegend; Cat. 325624) and cultured in macrophage differentiation media (RPMI1640 + 20% FBS + 1% P/S + 20 ng/mL M-CSF) for 6 days and 30,000 cells seeded overnight in a new plate. On day 7, 60,000 RAJI-GFP cells (target cells) were preincubated with STI-6643 or rituximab for 30 minutes at 37°C followed by addition to the plate containing the macrophages and incubated for 1 hour. Macrophages were labelled with anti-human CD11b PE (BioLegend; Cat. 982606) for 15 minutes prior the end of the incubation. Phagocytosis of target cells were measured as percent CD11b^+^GFP+ macrophages on Attune flow cytometer.

Alternately, Cell Proliferation Dye eFluor™ 670 (eBioscience; Cat. 65-0840-85) was used to label MDA-MB-231-Fluc target cells and RAJI-GFP were used without prior labelling. MDA-MB-231 cells were incubated for 2 h at 37°C prior to adding anti-CD47 (STI-6643 or Hu5F9) or anti-PD-L1 mAbs for 30-minutes. Then, PBMCs were added and incubated for 90 minutes at 37°C. After incubation, cells were resuspended in FACS buffer containing anti-human CD14-PE (BioLegend, Cat. 325606) antibody and incubated for 15 minutes at 4°C, washed, fixed, and analyzed by flow cytometry as above.

### Tumor Binding Assays of Anti-CD47 Antibodies

30,000 RAJI, 20,000 RAJI-GFP, OSCA-40 or OSCA-78 cells or 10,000 MDA-MB-231-Fluc cells per well were pre-incubated with anti-CD47 IgG_4_ antibodies (STI-6643 or Hu5F9) or isotype IgG_4_ control (1:3 serial dilutions starting at 100 µg/mL or 1:5 serial dilution starting at 300 µg/mL) and incubated for ~25 minutes at 37°C. Cells were then washed and resuspended in 50 µL/well of FACS buffer at RT containing APC-labelled anti-human IgG Fc antibody (BioLegend; Cat. 409306) diluted at 1:200 and incubated for 20 minutes at 37°C. Cells were washed and fixed in fixation buffer (BioLegend; Cat. 420801) and acquired on flow cytometry.

### Hemagglutination Assay

Peripheral blood obtained from healthy human donors, cynomolgus monkeys or Beagle dogs were washed with PBS 1X twice to make a 0.5% working solution. 50 µL of the working solution was distributed to each well of a U-bottom 96-well plate. Serially diluted anti-CD47 antibodies (clones STI-6643, Hu5F9, AO-176 and 13H3) or an isotype IgG_4_ control were prepared in PBS1X, 100 µL were distributed in the 96-well plate containing RBCs and gently mixed with a multichannel pipet. The plate was placed into a tissue culture incubator (5% CO2, 37°C) and photographed after ~ 20-hour incubation.

### 3-Way Mixed Lymphocyte Reaction

Freshly purified PBMCs from three different donors were mixed in equivalent proportions and plated at 0.5E+06 cells/well in 200 µL of RPMI1640 10% human AB serum (Valley Biomedical, Cat. HP1022) + P/S antibiotic at 37°C in a flat-bottom 96-well plate in the presence of isotype IgG_4_ or anti-CD47 mAbs clones STI-6643, Hu5F9, AO-176 or 13H3 at final concentrations ranging from 1 ng/mL to 100 µg/mL. After a 6-day incubation at 37°C, cells were washed and stained for 30 minutes at 4°C with the following antibodies mixture: anti-human CD4-PE (BioLegend, Cat. 317410), anti-human CD8-FITC (BioLegend, Cat. No. 300906), anti-human CD19-APC-Cy7 (BioLegend, Cat. 363010), and CD56-PB (BioLegend, Cat. 318326). Cells were washed then resuspended in 100 µL of fixation buffer (BioLegend; Cat. No. 420801) for 20 minutes at RT. 100 µL of FACS buffer at 4°C was added without washing and samples immediately analyzed by flow cytometery.

### SEB Assay

Fresh human PBMCs were plated at 2.0E+05 cells/well in U-bottom plates in 100 µL of complete RPMI 1640 medium. Isotype or anti-CD47 mAbs were added at a final concentration ranging from 1 ng/mL to 100 µg/mL (50 µL/well). Polyclonal T cell activation was triggered by addition of SEB (Staphylococcal Enterotoxin B, List Biological labs; Cat. 122) at a 25 ng/mL final concentration. The plate was incubated for 3 days at 37°C, supernatants collected. Cells were stained for 20 minutes at 4°C with 80 µl of an Ab mixture containing CD4-BV421 (dilution 1:200; Biolegend; Cat. 317434), CD8-PE (dilution 1:200; Biolegend; Cat. 344706) and CD25-AF647 (dilution 1:200; Biolegend; Cat. 356128), 2x washed and analyzed on flow cytometer. IFN-γ cytokine levels were measured in the supernatants by using the proinflammatory panel 1 (human) kit (Meso Scale Discovery; Cat. K15049D) as per manufacturer’s instructions.

### *In Vivo* Xenograft Human Tumor Models

#### Disseminated RAJI-Fluc Burkitt’s Lymphoma Model

On day 0 each Fox Chase/SCID mouse was injected IV into the tail vein with 1.0E+06 RAJI-Fluc cells in 200 µL of HBSS 1X. On day 7 mice were randomized and treated with anti-CD47 (clones STI-6643 or Hu5F9), rituximab, daratumumab or isotype control IgG_4_ antibodies at doses ranging from 0.01 to 60 mg/kg (as indicated for each experiment) by systemic IV injections (200 µL/mouse), 3x/week for 2 consecutive weeks. The body weight of each mouse was taken on day 0 and twice a week until termination of the study. Systemic tumor growth was evaluated on each animal on day 7 and 14, then twice a week until study termination by *in vivo* imaging using IVIS Lumina III Imaging System (PerkinElmer; CLS136334). Mice were euthanized as soon as first signs of hind leg paralysis otherwise mice were euthanized at study termination.

#### Solid MDA-MB-231 Breast Tumor Model

On day 0 NSG-Tg(Hu-IL15) mice were injected intraperitoneally (I.P.) with 4.0E+06 hPBMCs cells in 100 µL of sterile 1X PBS per mouse. On day 6 to 10, NSG-Tg(Hu-IL15) mice were injected subcutaneously (S.C.) with 5.0E+06 MDA-MB-231 cells in 100 µL 1X PBS per mouse. When tumor volume reached 50-100 mm^3^, mice were randomized and treated S.C. (distant from the tumor implantation site) with anti-CD47 or isotype control mAbs at 10 mpk as described in **Supplementary Figure 6A** Tumor growth was assessed at least twice a week by measuring tumor volumes using the formula V=(Length×Width^2^)/2. Mice were immediately euthanized if tumor reached 2,000 mm^3^. Body weights were recorded on day 0 (baseline) and then once a week until termination of the study. Blood samples were collected before mAb treatment (day -1), then one day after the last treatment and at take down, to assess the impact of Ab treatment on circulating T-cells by flow cytometry analysis.

### *In Vivo* Xenograft vs Host Disease (GVDH) Model

To induce GVDH on day 0 NSG-Tg(Hu-IL15) mice were injected I.P. with 10E+06 hPBMCs cells in 100 µL of DPBS (1X) per mouse. On day 10 peripheral blood T cell engraftment was checked and mice randomized into 3 groups based on % of CD3^+^CD45^+^ cells and treated with three doses of anti-CD47 mAb clones (STI-6643 or Hu5F9) or isotype control mAb (RSV IgG_4_) on days 11, 14 and 17 respectively. Antibodies were injected sub cutaneous at 10 mg/kg/dose (diluted in PBS). Depleting activity of CD47 antibodies was assessed in peripheral blood one day after last antibody dose (on day 19). Mice were monitored for the signs of GVHD sickness due to the lymphocytes expansion and body weights recorded.

## Data Availability Statement

The raw data supporting the conclusions of this article will be made available by the authors, without undue reservation.

## Ethics Statement

The animal study was reviewed and approved by the Study Review Committee at Sorrento Therapeutics. Experiments are conducted under ACUP# EB16-057 and EB17-010-018 in accordance with Explora BioLabs IACUC guidelines.

## Author Contributions

Conception and design, DB, YT, CP, JG, and AS. Development of methodology, DB, YT, CP, JG, IR, BS, and AS. Acquisition of data, IR, YT, CP, LR, JG, AS, DB, and HZ. Reagents and materials, HZ, BS, LK, and YZ. Animal work and *in vivo* data analysis, IR, LR-N, AS, CP, YT, and DB. Analysis and interpretation of data (statistical analysis, biostatistics analysis), YT, DB, IR, AS, and CP. Writing, review, and/or revision of the manuscript, YT, DB, and RA. Study supervision, DB, GK, HJ, YT, and CP. All authors approved the final version for publication and agreed to be accountable for all aspects of the work.

## Conflict of Interest

Authors YT, AS, LR-N, HZ, LK, YZ, JG, HJ, RA and DB were employed by Sorrento Therapeutics, Inc. IR is employed by Janssen, CP is employed by Turnstone Biologics and GK is employed by Oncternal Therapeutics. BS, IR, CP, GK were previously employed by Sorrento Therapeutics.

The remaining authors declare that the research was conducted in the absence of any commercial or financial relationships that could be construed as a potential conflict of interest.

## Publisher’s Note

All claims expressed in this article are solely those of the authors and do not necessarily represent those of their affiliated organizations, or those of the publisher, the editors and the reviewers. Any product that may be evaluated in this article, or claim that may be made by its manufacturer, is not guaranteed or endorsed by the publisher.
