# Supplementary material for: A Novel Affinity Engineered Anti-CD47 Antibody With Improved Therapeutic Index That Preserves Erythrocytes and Normal Immune Cells
Source: Front Oncol. 2022 May 19;12:884196. doi: 10.3389/fonc.2022.884196 (PMC9161735; doi:10.3389/fonc.2022.884196)

Supplementary Figure 1

a

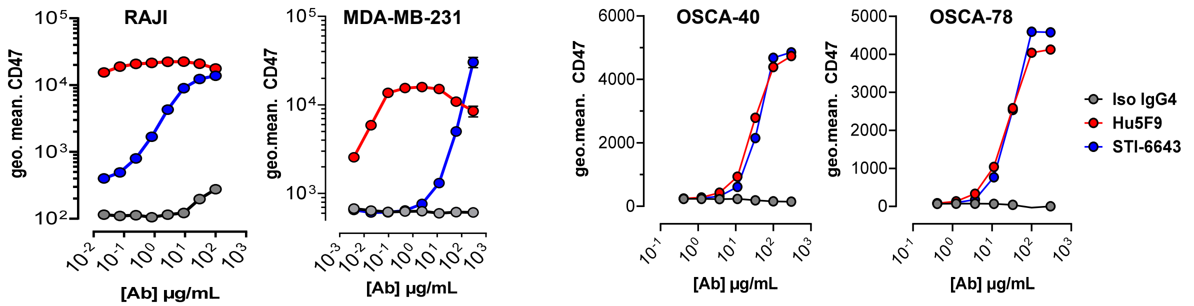

EC50 values (µg/mL)	Isotype IgG4	STI-6643	Hu5F9
RAJI	NM	6.638	NM
MDA-MB-231	NM	NM	NM
OSCA-40	NM	34.9	26.5
OSCA-78	NM	27.6	22.7

NM=Not measurable (no full dose-response curve to accurately calculate EC50)

b

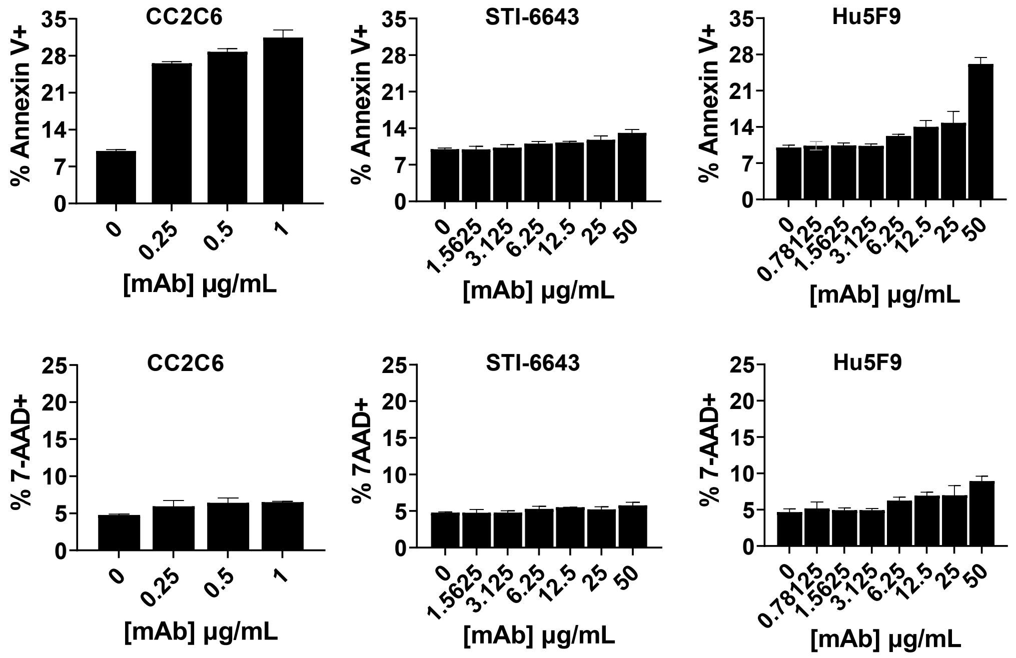

Supplementary Figure 2

a

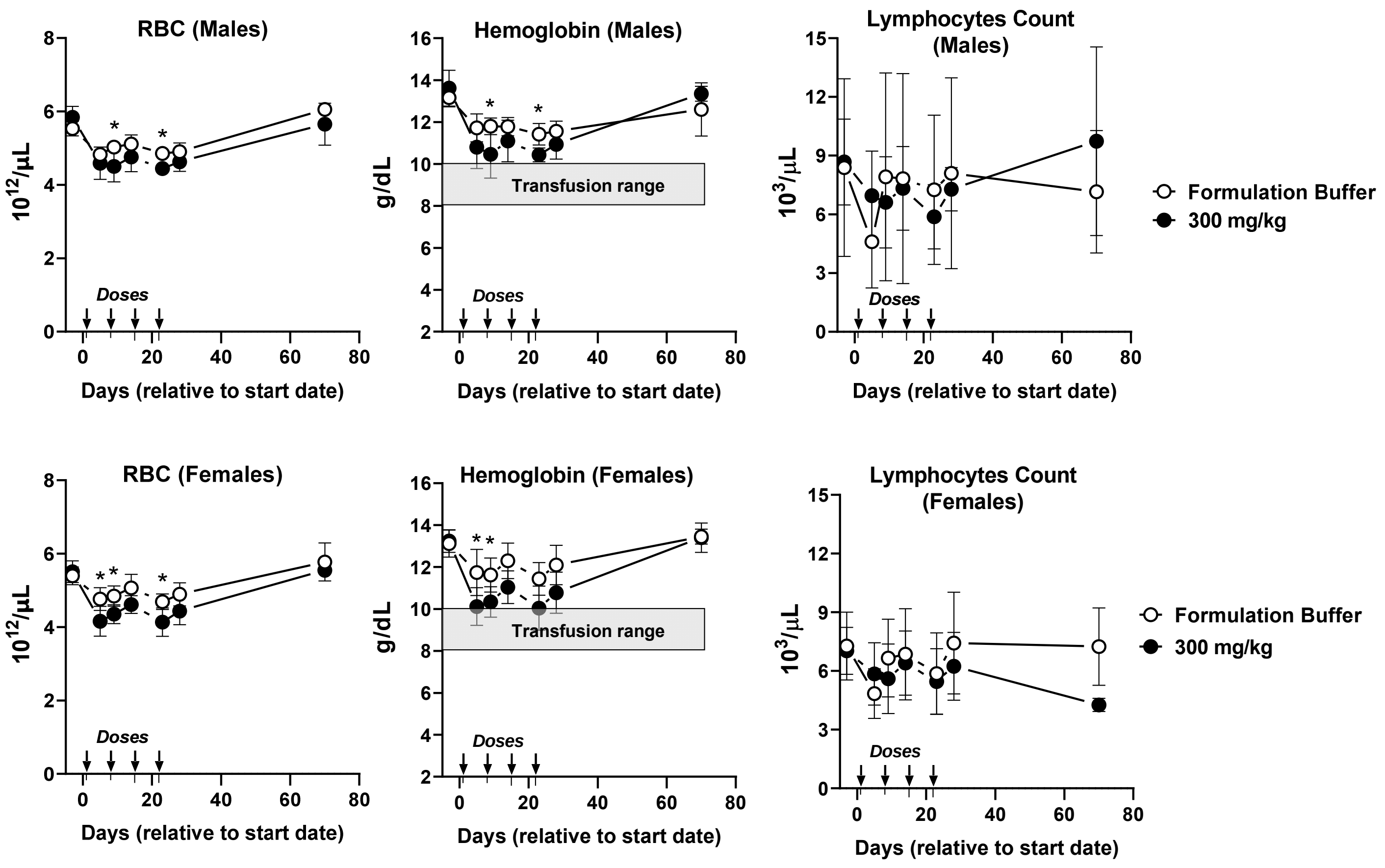

b

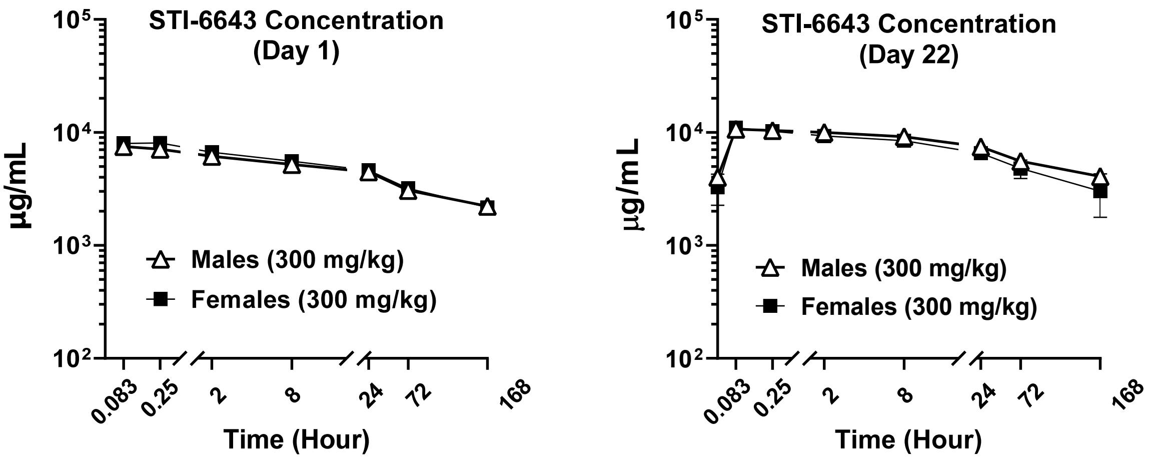

c

Gender	Date	Number of Animals	Dose	T_{max}			C_{max} (µg/mL)			$AUC_{(0-168 h)}$ (h*µg/mL)			T1/2 (Hours)		
			(mg/kg)	Median	Min	Max	Mean	±	SD	Mean	±	SD	Mean	±	SD
Female	D1	5	300	2	2	2	6685.86	±	452.08	571136.90	±	43665.05	102	±	0
	D22	5	300	2	2	8	9351.43	±	960.26	835329.19	±	146799.64	56.7	±	0
Male	D1	5	300	2	2	8	6162.70	±	775.91	552376.17	±	43909.21	NC	±	NC
	D22	5	300	2	2	2	10012.39	±	455.76	981030.99	±	109375.75	NC	±	NC

NC: Not calculable (the concentration of STI-6643 in Cynomolgus monkey plasma has not reached the elimination phase, and the accurate half-life cannot be calculated).

Supplementary Figure 3

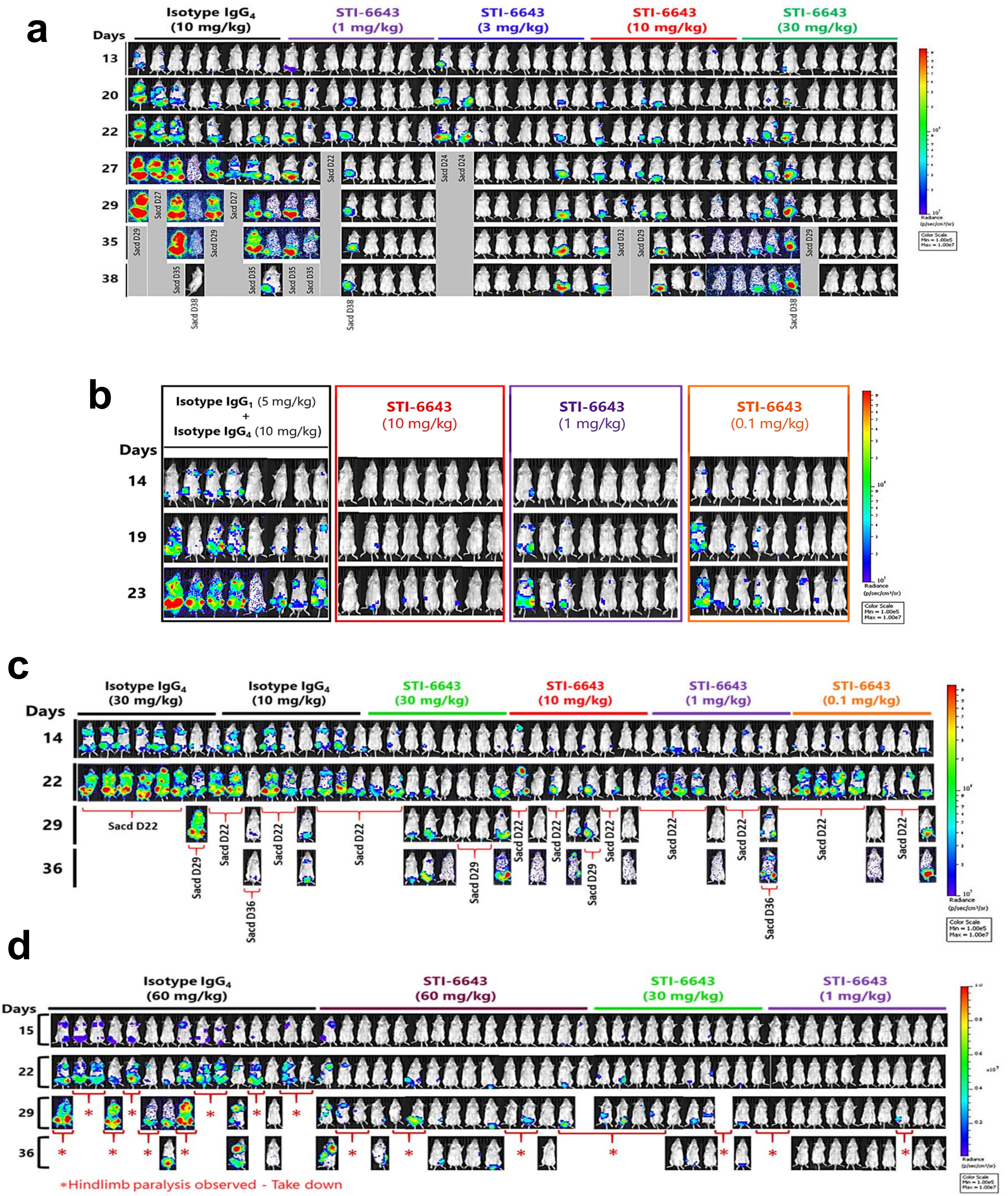

Supplementary Figure 4

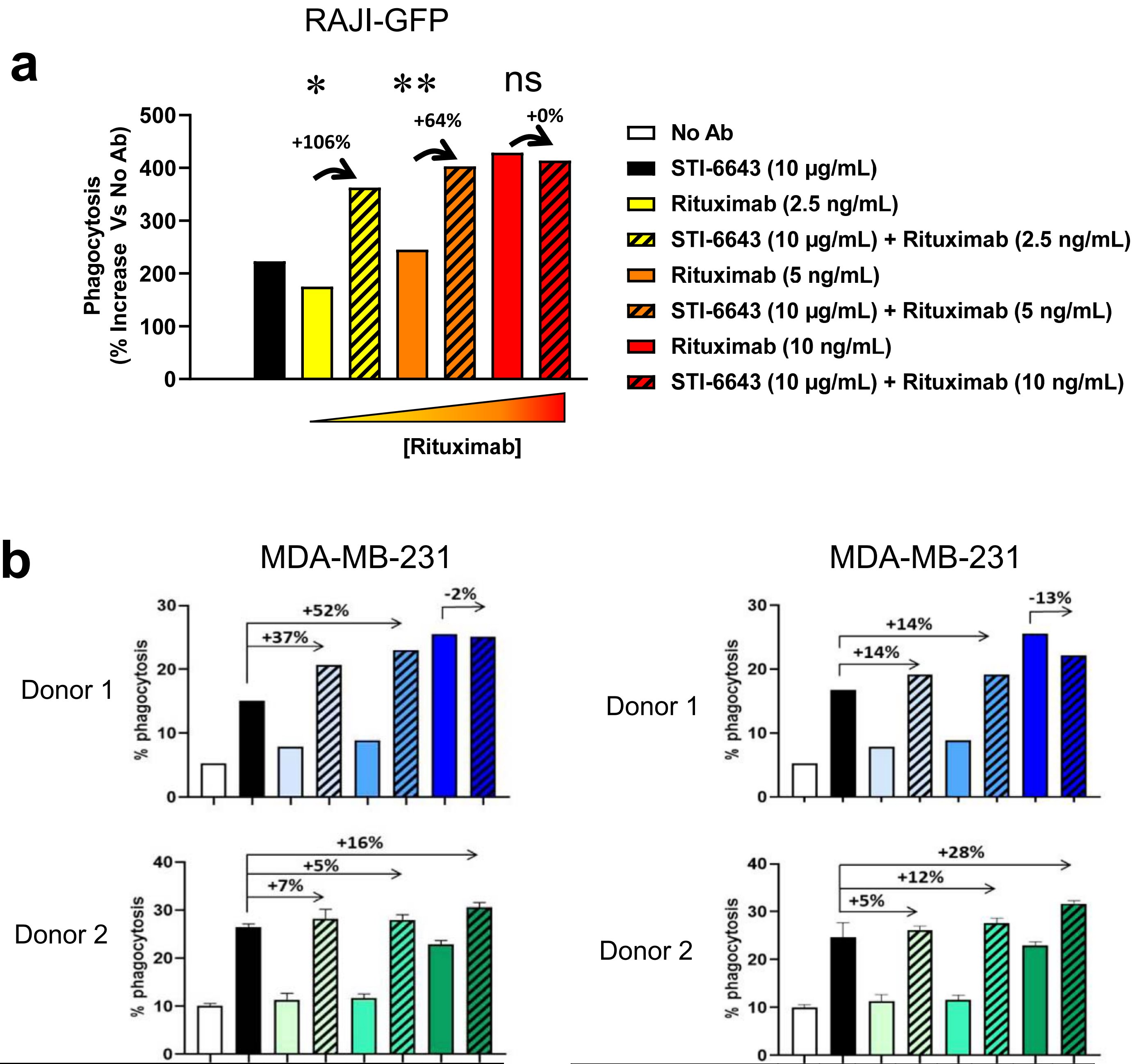

STI-6643 (10 µg/mL)		+		+		+		+
Avelumab (0.01 ng/mL)			+	+				
Avelumab (0.1 ng/mL)					+	+		
Avelumab (1 ng/mL)							+	+

STI-6643 (1 µg/mL)		+		+		+		+
Avelumab (0.01 ng/mL)			+	+				
Avelumab (0.1 ng/mL)					+	+		
Avelumab (1 ng/mL)							+	+

Supplementary Figure 5

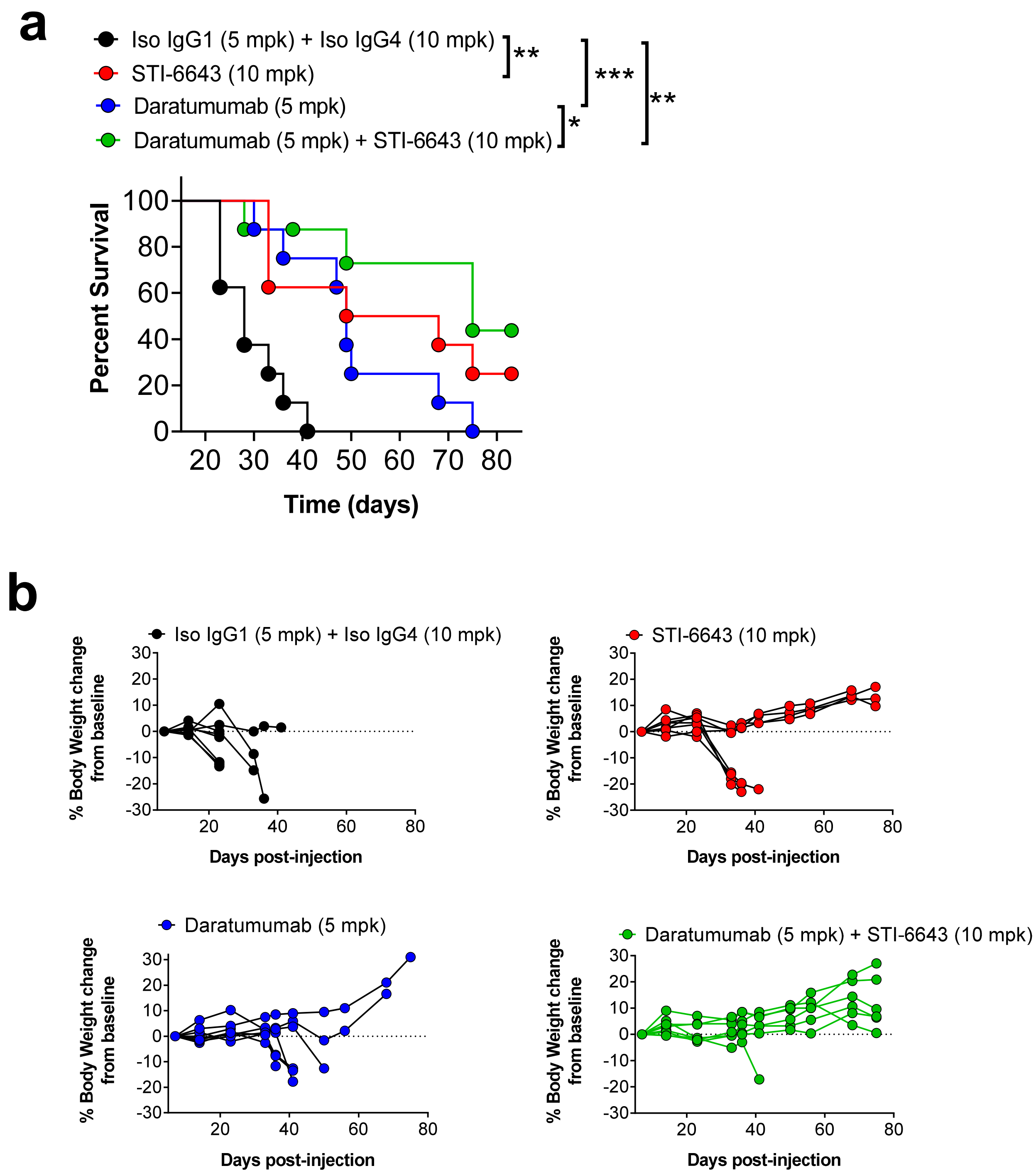

Supplementary Figure 6

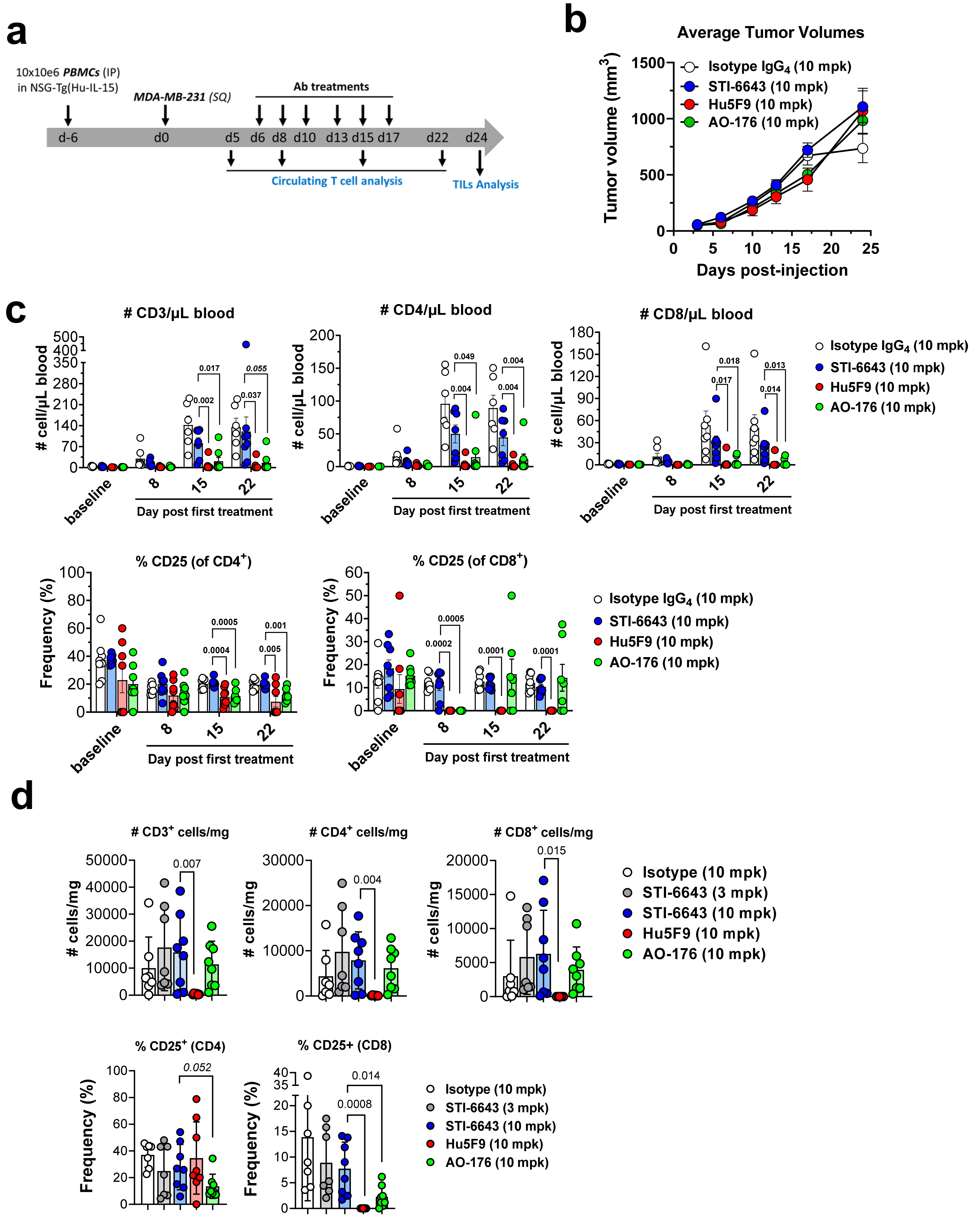

Supplement: Supplementary file 1 [file DataSheet_1.pdf]
