# Supplementary material for: A Novel Affinity Engineered Anti-CD47 Antibody With Improved Therapeutic Index That Preserves Erythrocytes and Normal Immune Cells
Source: Front Oncol. 2022 May 19;12:884196. doi: 10.3389/fonc.2022.884196 (PMC9161735; doi:10.3389/fonc.2022.884196)
Supplement: Supplementary file 2 [file DataSheet_2.pdf]

Supplementary Methods

Clone selection for fully human anti-CD47 IgG4-S228P antibody clone STI-6643.

The pXC-18.4 and pXC-17.4 combined double gene expression vector was linearized and electroporated into Lonza Chinese Hamster Ovary (CHO) K1SV GS-KO host cells and the transfected cells were expanded first in 48-well and then 24-well plates as recommended by the manufacturer. Each clone was analyzed for antibody titer by OCTET Red Protein A and the top 30 clones were evaluated in a 13-day fed-batch culture and titers re-checked. From the top 10 producers, three cell line clones were generated and the highest IgG antibody producer over 20 passages was deemed the most stable and selected as the top cell line to proceed with the future manufacturing processes.

Cloning and expression of human anti-CD47 reference clones (Hu5F9, AO-176/VLX09-hum06 and TJC4/13H3).

The human heavy (VH) and light (VL) chain variable domains of each antibody were obtained from publicly available sources (Hu5F9-G4[1] and patent WO2017/049251 A3 for AO-176[2] and patent WO2018/075857 A1 for TJC4/13H3). DNA fragments encoding VH and VL domain sequences were chemically synthesized and cloned into Sorrento Therapeutics' proprietary HC and LC two vector expression systems. The antibodies were expressed in the Free Style CHO-S Cells (Thermo Fisher Scientific; Cat. No. R80007) in CHO-S-SFM media with hypoxanthine and thymidine (Life Technologies; Cat. No. 12052098). Each clone was purified using the protein A MabSelect SuReLX resin (Cytiva; Cat. No. 1754702). Hu5F9 and AO-176 antibodies were polished by Capto S ImpAct resin (Cytiva; Cat. No. 17544110) while 13H3 clone was polished using HiLoad 26/600 Superdex 200pg column (Sigma Aldrich; Cat. No. 28-9896-36). Antibodies were stored in formulation buffer (20 mM Histidine, 100 mM NaCl, 120 mM Sucrose; pH = 5.8).

Kinetic Characterization Using BIACORE

Anti-human Fc antibody was immobilized on a CM5 sensor chip to approximately 8,000 resonance units (RU) using standard N hydroxysuccinimide/N Ethyl-N′-(3-dimethylaminopropyl) carbodiimide hydrochloride (NHS/EDC) coupling methodology. STI-6643 at 1.5 μg/mL was captured for 60 seconds at a flow rate of 10 μL/minute. Recombinant human, cynomolgus or canine CD47/His (ACROBiosystems) proteins were serially diluted in a running buffer of 0.01 M

HEPES pH 7.4, 0.15 M NaCl, 3 mM (EDTA), 0.05% v/v Surfactant P20 (HBS EP+). All measurements were conducted in HBS-EP+ buffer with a flow rate of 30 µL/minute. A 1:1 (Langmuir) binding model was used to fit the data. Assay was performed at 25 °C.

Cell Death Assay

Viability of CCRF-CEM cells was >95% at the time of assay. In a low evaporation 96-well U-bottom plate (Costar; Cat. 3799), anti-CD47 mAb clones STI-6643, CC2C6 (BioLegend, Cat. 323102 and contains 0.09% sodium azide) and Hu5F9 were added at a 2x final concentration diluted in media followed by addition of 50,000 CCRF-CEM cells/well in 50 µL of complete media making final volume to 100 µL/well. Plates were incubated at 37 °C for 24 h. Cells were harvested and washed twice with cold 1X Annexin V binding buffer (BioLegend, Cat. 422201) followed by staining with FITC Annexin V (1:50 dilution) and 7-AAD (1:25 dilution) for 15 minutes at RT in dark. Without washing 80 µL Annexin V was added to each well and immediately acquired on Attune NxT flow cytometer. Data were analyzed by using FlowJo and GraphPad Prism software.

Toxicokinetics (TK) in non-human primates

Animals were socially-housed in stainless-steel cages. Primary enclosures were as specified in the USDA Animal Welfare Act (9 CFR, Parts 1, 2, and 3) and as described in the *Guide for the Care and Use of Laboratory (Institute of Laboratory Animal Resources (U.S.). Committee on Care and Use of Laboratory Animals ;National Research Council (U.S.). Committee for the Update of the Guide for the Care and Use of Laboratory Animals}.* Animals were housed at a temperature of 18 to 29 °C, a humidity of 30% to 70%, and a 12-hour light/dark cycle was maintained (except during designated procedures). Eight or greater air changes per hour with 100% fresh air (no air recirculation) were maintained in the animal rooms.

For the dose-finding study IV bolus injection once weekly (on days 1, 8, 15, and 22) for a total of 4 doses to cynomolgus monkeys (n=4 per group; 2 males and 2 females) at a dose level of 0, 30, 90 or 150 mg/kg/dose (dose volume of 5 mL/kg for all groups). Animals underwent a 28-day recovery period before being necropsied on Day 57. Clinical pathology looking at hemoglobin concentration, RBC and lymphocyte counts were conducted between d7 and d57 relative to first antibody infusion. For blood coagulation and clinical chemistry blood samples were collected on

d7 and d29 relative to first antibody infusion. For Pharmacokinetic analysis, blood was collected and processed to plasma at 0 min, 5 min, 15 min and 2, 8, 24, 72 and 168 h. Plasma STI-6643 levels were measured using a validated ELISA method with a dynamic range of 0.2 to 5 µg/mL. TK parameters were estimated using Phoenix WinNonlin v1.4 pharmacokinetic software (Certara, USA)

For the high-dose study STI-6643 when given by IV bolus injection once weekly (on days 1, 8, 15, and 22) for a total of 4 doses to cynomolgus monkeys (n=10 per group; 5 males and 5 females) at a dose level of 0 and 300 mg/kg/dose (dose volume of 10 mL/kg for all groups). Animals underwent a 6-week recovery period before being necropsied on Day 71. Clinical pathology looking at hemoglobin concentration, RBC and lymphocyte counts were conducted between d7 and d28 relative to first antibody infusion and analyzed by the ADVIA 2120 Automated Hematology Analyzer. For coagulation and clinical chemistry blood samples were collected at -7 and 28 days relative to first antibody infusion and analyzed by the Sysmex CS-5100 Automated Coagulation Analyzer and Hitachi-7180 Automatic Clinical Analyzer, respectively.

Pharmacokinetic (PK) analysis was conducted in male and female cynomolgus monkey plasma on days 1 and 15, following once weekly slow IV bolus injection of STI-6643 at dose levels of 30, 90 and 150 mg/kg/dose. Blood was collected (on days 1 and 15) and processed to plasma at 0 min (pre-dosing), 5 min, 15 min and 2, 8, 24, 72 and 168 h. Plasma STI-6643 levels were measured using a validated ELISA method with a dynamic range of 0.1 to 3.2 µg/mL. TK parameters were estimated using Phoenix WinNonlin v6.4 pharmacokinetic software (Certara, USA) using a non-compartmental approach consistent with the IV (slow bolus) route of administration. All parameters were generated from STI-6643 individual concentrations in plasma from Days 1 and 22. The primary toxicokinetic parameters T_{max}, C_{max} and $AUC_{(0\text{-}168\ h)}$ were calculated using Watson LIMS 7.4.2 software with at least two plasma concentrations values above lower limit of quantification (LLOQ) and reported as Median (Min and Max), Mean ± SD and Mean ± SD, respectively. $T_{1/2}$ was calculated using Phoenix WinNonlin v6.4 software and reported as Mean ± SD. SPSS Statistics 21 was used to conduct t-test analysis for gender differences. When both *p* value was <0.05 and exposure (C_{max} and $AUC_{(0\text{-}168\ h)}$) ratio was outside a 0.5-2.0 range, data were considered statistically different between genders. Plasma concentration below the lower limit of quantitation (0.1 µg/mL) was considered as below the quantification limit (BQL). If the

concentrations of more than half of the samples were below LLOQ at the same time point, data were excluded from the average calculation, reported as "NC."

Data and statistical analysis

Bioluminescence imaging data: Bioluminescence was quantified using the Living Image® software from PerkinElmer. The radiance (p/sec/cm^2/sr) was set between 1.0E+05 and 5.0E+06 for each analysis and bioluminescence picture. Tumor growth data are given in total flux (p/s). When data of at least two groups were compared over time (bioluminescence values and anti-CD47 serum concentrations) the mixed-effects analysis for multiple comparisons were used: Two-way ANOVA where each row represents a different time point (so match values are spread across a row), comparing column means (main column effect) and corrected for multiple comparisons using the Tukey's comparisons test, with individual variances computed for each comparison. Log-rank tests are used to assess statistical significance in survival data. All statistical tests were 2-sided, and results were considered statistically significant at $P < 0.05$. Outliers were identified and excluded using the ROUT method (at Q=1%) in the GraphPad Prism software.

For the dose-finding TK study: Statistical analysis was performed by using pooled males and females monkeys for each group: For data collected and/or reported in Provantis™ (in-life (clinical observations, body weights, food consumption); clinical pathology (clinical chemistry, coagulation, hematology); postmortem (organ weights, necropsy, histopathology)), Levene's test was used to assess the homogeneity of group variances parametric assumption at the 5% significance level. Datasets with at least 3 groups were compared using an overall 1-way ANOVA F-test or Kruskal-Wallis test (if parametric assumptions were not met) at the 5% significance level. The above pairwise comparisons were conducted using a 2-sided Dunnett's or Dunn's test, respectively, if the overall test was significant. All significant pairwise comparisons were reported at the 1 and 5% significance levels. For data external to the Provantis™ system, the assumptions that permitted use of a parametric ANOVA were verified using the Shapiro-Wilkes test for normality of the data and Levene's test for homogeneity of variance, with $p \leq 0.001$ level of significance required for either test to reject the assumptions. If both assumptions were fulfilled, a single-factor ANOVA was applied, with animal grouping as the factor, utilizing a $p \leq 0.05$ level of significance. If the parametric ANOVA was significant at $p \leq 0.05$, Dunnett's test was used to identify statistically significant differences between the control group and each test article dosed

group at the 0.05 level of significance. If either of the parametric assumptions was not satisfied, the Kruskal-Wallis non-parametric ANOVA procedure was used to evaluate intergroup differences ($p \leq 0.05$). The Dunn's multiple comparison test was applied if this ANOVA was significant, again utilizing a significance level of $p \leq 0.05$.

For the high-dose TK study: Statistical analysis was performed by Provantis system. Comparison between test article-treated groups and the vehicle control group was performed using the following statistical methods: (1) Data within groups were evaluated for homogeneity of variance by Levene's test. For data whose variances were homogeneous ($p>0.05$), a one-way analysis of variance (ANOVA) was performed on the data; for nonhomogeneous data ($p\leq0.05$), a logarithmic transformation was automatically applied to obtain log data, and a Levene's test was applied to the log data again. For log data whose variances were homogeneous ($p>0.05$), a one-way analysis of variance (ANOVA) was performed on the log data; for nonhomogeneous log data ($p\leq0.05$), a rank transformation was applied on the log data to obtain rank data before Kruskal-Wallis test being performed. (2) Differences between the test article-treated groups and the vehicle control group were further tested by Dunnett t-test for pairwise comparisons (at the 0.05 and 0.01 levels) only when the ANOVA was significant ($p\leq0.05$); Otherwise, no further analyses were performed. (3) When significant results were obtained in the Kruskal-Wallis test ($p\leq0.05$), Dunnett t-test on rank data was used for pairwise comparisons between the test article-treated groups and the vehicle control group (at the 0.05 and 0.01 levels); When no significant results were obtained in the Kruskal-Wallis test ($p>0.05$), no further analyses were performed.

1. Liu J, Wang L, Zhao F, Tseng S, Narayanan C, Shura L*, et al.* Pre-Clinical Development of a Humanized Anti-CD47 Antibody with Anti-Cancer Therapeutic Potential. *PLoS One* 2015; **10**(9)**:** e0137345.

2. Puro RJ, Bouchlaka MN, Hiebsch RR, Capoccia BJ, Donio MJ, Manning PT*, et al.* Development of AO-176, a Next-Generation Humanized Anti-CD47 Antibody with Novel Anticancer Properties and Negligible Red Blood Cell Binding. *Mol Cancer Ther* 2020 Mar; **19**(3)**:** 835-846.

Supplementary Figure Legends

Supplementary Figure 1. (**a**) Binding of anti-CD47 antibodies (clones STI-6643 and Hu5F9) to a human B cell lymphoma cell line (RAJI), human breast cancer cell line (MDA-MB-231) and two canine osteosarcoma cell lines (OSCA-40 and OSCA-78). Isotype matching antibody IgG_4 was used as negative control. EC50 values are provided in the Table. NM, not measurable. (**b**) Cell killing was assessed at 24 h by staining uptake for Annexin-V (top panels) and 7-AAD (bottom panels) upon treatment with anti-CD47 clones CC2C6, STI-6643 and Hu5F9 (left, middle and right panels respectively). Data is representative of 1 experiment in CCRF-CEM cells and more than 3 experiments in RAJI cells (data not shown).

Supplementary Figure 2. High dose (300 mg/kg) toxicokinetic study in non-human primates. Individual cynomolgus monkeys (five males and five females) were administered intravenously on days 1, 8, 15 and 22 with STI-6643 at 300 mg/kg or the same volume of formulation buffer (marked by arrows). The hematological parameters of individual monkeys were monitored for 11 weeks. (**a**) The RBC, hemoglobin levels, and lymphocytes counts were measured overtime for both males and females. The shaded bar indicates the range of hemoglobin that might trigger transfusion in humans. (**b**) Serum STI-6643 levels were determined after a single (PK day 1) or the fourth infusion (PK day 22) as indicated. (**c**) Pharmacokinetic parameters in cynomolgus monkeys dosed in panel b. T_{max}: time of C_{max}, C_{max}: maximum observed concentration, $AUC_{(0-168h)}$: area under the curve and $T_{1/2}$: half-life. Data are given as a mean ± SD. *$p \leq 0.05$ when comparing formulation buffer and 300 mg/kg groups at the indicated time point.

Supplementary Figure 3. (**a-d**) Bioluminescence images of individual mice captured at different time points post tumor cell implantation treated with various doses of isotype or STI-6643 antibodies as indicated on the figures. Mice with signs of hind leg paralysis were immediately sacrificed (sacd) and are indicated for each study. Data from four independent experiments are shown; dosages may vary from experiment to experiment and are indicated on the figures.

Supplementary Figure 4. (**a**) Phagocytosis of RAJI-GFP cells by CD14+-derived human macrophages in the presence of 10 µg/mL of STI-6643 IgG_4 used alone or in combination with variable concentrations of anti-CD20 IgG_1 (rituximab) ranging from 2.5 to 10 ng/mL. (**b**) Phagocytosis of eF670 dye-labelled MDA-MB-231 cells by $CD14^+$ PBMCs in the presence of 1 µg/mL (right panel) or 10 (left panel) µg/mL of STI-6643 alone or in combination with anti-PD-L1 IgG_1 (avelumab) at various concentrations ranging from 0.01 to 1 ng/mL, conducted with two different donors (top versus bottom panels) used to generate human macrophages. The percent increase between 'anti-CD47 + anti-PD-L1' combination and the most efficient single mAb treatment alone are shown on the graph. Data are representative of more than three independent experiments. Statistical significance assessed by Student's *t*-test (not significant [ns] $p > 0.05$, $*p \leq 0.05$, $**p \leq 0.01$).

Supplementary Figure 5. (**a**) Kaplan-Meier survival curves of animals treated with anti-CD47 IgG_4 (clone STI-6643), anti-CD38 IgG_1 (daratumumab) used alone or in combination and isotype controls (IgG_1 and IgG_4 isotypes in combination) in RAJI-Fluc disseminated xenograft model. Statistical significance was assessed using the log-rank Mantel-cox test; Results were deemed significant at a level of $p<0.05$ ($*p \leq 0.05$, $**p \leq 0.01$, $***p \leq 0.001$). (**b**) Body weight as a percent of baseline at the start of experiment in various treatment groups. Data are representative of three independent experiments.

Supplementary Figure 6. STI-6643 preserved human T cells in the tumor microenvironment in a solid xenograft tumor model. (**a**) A schematic of the experimental procedure. (**b**) Tumor volumes for each treatment group. Data are presented as a mean ± SEM of 7-8 mice per group. (**c**) Analysis of circulating human $CD3^+$ T cells/µL of mouse blood showing total counts of $CD3^+$ (top left panel), $CD4^+$ (top middle panel) and $CD8^+$ (top right panel) T cells at baseline (d5, before antibody treatment) and on days 8, 15 and 22 post tumor cell implantation. The frequency of activated $CD25^+$ T cells within the CD4+ (bottom left panel) and $CD8^+$ (bottom right panel) T cell compartment is also shown for each treatment group. (**d**) Tumor-infiltrating T cells per mg of tumor tissue (total CD3 [top left panel], total CD4 [top middle panel], total CD8 [top right panel], $CD4^+CD25^+$ [bottom left panel] and $CD8^+CD25^+$ [bottom right panel]) at study take-down (day 24) in mice treated with anti-CD47 antibody clone STI-6643 or reference AO-176 and Hu5F9

clones or isotype control. Data are representative of two independent experiments. Statistical significance assessed by two-tailed Welch's t-test; Results were deemed significant at a level of $p<0.05$.
